# Erratum for Niu et al., “Understanding the Linkage between Elevation and the Activated-Sludge Bacterial Community along a 3,600-Meter Elevation Gradient in China”

**DOI:** 10.1128/aem.00444-26

**Published:** 2026-03-31

**Authors:** Lihua Niu, Yi Li, Peifang Wang, Wenlong Zhang, Chao Wang, Qing Wang

## ERRATUM

Volume 81, no. 19, p. 6567–6576, 2015, https://doi.org/10.1128/aem.01842-15. Figure 1 should appear as shown in this erratum. Sample site CC should be labeled sample site JL, and the southern region of China should be included in the map.

**Fig 1:**
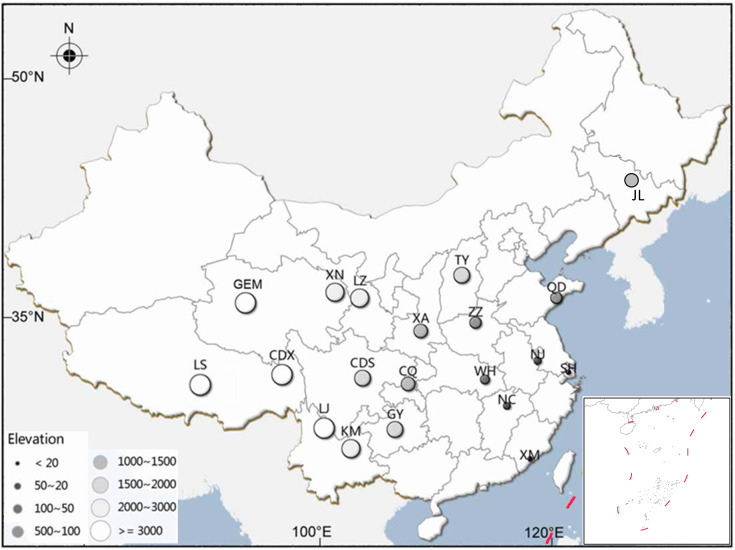


Figure 2: Sample site CC should be labeled sample site JL.

Figure 3: Sample site CC should be labeled sample site JL.

Figure 7b: Sample site CC should be labeled sample site JL.

